# Two Reasoning Strategies in Patients With Psychological Illnesses

**DOI:** 10.3389/fpsyg.2019.02335

**Published:** 2019-10-22

**Authors:** Amelia Gangemi, Katia Tenore, Francesco Mancini

**Affiliations:** ^1^Dipartimento di Scienze Cognitive, University of Messina, Messina, Italy; ^2^Scuola di Psicoterapia Cognitiva, Rome, Italy

**Keywords:** hyper-emotion theory, emotions, reasoning, anxiety disorders, obsessive-compulsive disorders, corroboratory strategy, refutatory strategy

## Abstract

Hyper-emotion theory states that psychological disorders are conditions in which individuals experience emotions that are appropriate to the situation but inappropriate in their intensity. When these individuals experience such an emotion, they are inevitably compelled to reason about its cause. They therefore develop characteristic strategies of reasoning depending on the particular hyper-emotion they experience. In anxiety disorders (e.g., panic attack, social phobia), the perception of a disorder-related threat leads to hyper-anxiety; here, individuals’ reasoning is corroboratory, adducing evidence that confirms the risk (*corroboratory strategy*). In obsessive-compulsive disorders, the threat of having acted in an irresponsible way leads to both hyper-anxiety and guilt; here, individuals’ reasoning is refutatory, adducing only evidence disconfirming the risk of being guilty (*refutatory strategy*). We report three empirical studies corroborating these hypotheses. They demonstrate that patients themselves recognize the two strategies and spontaneously use them in therapeutic sessions and in evaluating scenarios in an experiment.

## Introduction

The maintenance of psychological illnesses and their resistance to change have a paradoxical nature: people who worry about a certain catastrophe continue to experience fear despite the evidence of their survival. Cognitive models of psychopathology focus their attention on the dysfunctional beliefs implicated in the genesis and maintenance of these illnesses (e.g., Beck, 1976; Harvey et al., 2004; Johnson-Laird et al., 2006). The hyper-emotion theory of psychopathology is in line with these cognitive models (Johnson-Laird et al., 2006; Mancini et al., 2007). Such a model states that psychological disorders are conditions in which individuals experience emotions that are appropriate to the situation but inappropriate in their intensity. The hyper-emotion model is based on a cognitive view where the emotions are related to conscious or unconscious evaluations. These evaluations predispose individuals to certain thoughts and actions (Oatley and Johnson-Laird, 1987). Hence, when individuals are experiencing a hyper-emotion, they are inevitably compelled to reason about its cause, and, over the long term, their ability to reason in this way increases (cf. Gangemi et al., 2013). As a consequence, patients acquire specific reasoning strategies that depend on the hyper-emotion elicited by the disorder-related threat. The paradox is that these strategies serve to support the psychological disorders, leading as they do to the continued confirmation of the dysfunctional beliefs central to them. In anxiety disorders, such as hypochondria, panic attack, and social phobia, the perception of a disorder-related threat leads to hyper-anxiety. Prudent cognitive processes are thus oriented toward corroborating the danger in order to avoid it or prevent it, because it is “better to be safe than sorry” (e.g., de Jong et al., 1997, 1998; Smeets et al., 2000). For example, de Jong et al. (1998) showed that hypochondriac individuals were more likely to select confirming information when judging a danger related to a conditional hypothesis about physical health (e.g., *If a person suffers from a headache, then that person must have a brain tumor)*, and disconfirming information of the safety conditional hypothesis. The reasoning of anxiety disorder patients should therefore be corroboratory, adducing only evidence that prudentially confirms the risk. We refer to this as the “corroboratory” strategy.

In certain cases, such threats may be associated with the guilt emotion and responsibility, which have been proven to have a key role in the onset and maintenance of Obsessive-Compulsive Disorder (OCD; e.g., Shapiro and Stewart, 2011; Mancini and Gangemi, 2015). In fact, Shapiro and Stewart (2011) showed that: (1) in non-clinical samples, guilt determines obsessive-compulsive-like symptoms, together with an increased perception of threat (see Gangemi et al., 2007), over-responsibility, and intrusive thoughts/impulses (Niler and Beck, 1989); and, (2) in neuro-imaging studies of non-clinical samples, the state of guilt activates brain regions in proximity to OCD-affected regions (Shin et al., 2000; Takahashi et al., 2004). In obsessive patients, cognitive processes should therefore aim to exclude the possibility of guilt for having done something wrong from leading to, for example, risk of contamination. If one wants to falsify a risk with certainty, s/he can only try to imagine all the situations in which the condition could be true and falsify them one by one. Accordingly, obsessive patients should focus on all the possibilities that could put them at risk and then try to refute them beyond reasonable doubt. This strategy is chosen because it is not possible to act on the facts themselves, for example, by changing them (I cannot go back and avoid touching a contaminating photograph). In this case, not only the results obtained but also one’s own efforts are evaluated against very high standards. The ultimate goal of this strategy is to avoid the self-accusation of having not been up to fulfilling one’s duties. This goal has a paradoxical effect: it suggests possible mechanisms by which the risk could be real (see Johnson-Laird et al., 2006). The reasoning of obsessive-compulsive patients should therefore be refutatory, searching for evidence to refute the risk. We accordingly refer to this reasoning strategy as “refutatory.” Unlike the corroboratory strategies in anxiety disorders, so far, no studies have investigated the refutatory form of reasoning in obsessive patients. Only the obsessive-like step-by-step reasoning from a neutral situation toward an unlikely catastrophic consequence, examined by Giele et al. (2011) is comparable to the hypothesized refutatory pattern of reasoning. The obsessive-like step-by-step reasoning form would induce uncertainty and increase the perceived probability of a negative outcome. But, in their study, the authors did not evaluate whether participants, when engaging in this step-by-step reasoning, try to find counterexamples of the obsessive-like consequence, although it would be plausible that the experiment also induced some form of refutatory reasoning.

Here are two vignettes illustrating the contrasting types of reasoning strategies but using contents suggestive of hypochondria (in Johnson-Laird et al., 2006).

The first vignette illustrates corroboratory reasoning:

I’m afraid of the slight pain I feel in my abdomen on the same side as my liver. It could be a symptom of cancer, a liver cancer. I remember an uncle of mine who died from liver cancer after a lot of suffering. In the beginning, his symptoms were the same as mine: he had a similar stomach ache. He didn’t take any notice, and the doctors told him that he wasn’t ill. But meanwhile, the cancer was spreading. Now, in the same way, the cancer may be spreading in my abdomen. Moreover, it seems to me that I look unhealthy; my tongue is pasty; sometimes I have a bitter taste in my mouth. I look pale, and I could be anemic.

This second vignette illustrates refutatory reasoning:

I’m afraid of the slight pain I feel in my abdomen on the same side as my liver. It could be a symptom of cancer, a liver cancer. I remember an uncle of mine who died from liver cancer after a lot of suffering. But he was in his eighties, and a liver cancer at my age is not common. On the other hand, it’s not impossible. Moreover, it seems to me that I look unhealthy; my tongue is pasty; sometimes I have a bitter taste in my mouth. I look pale, and I could be anemic. Of course, these are common symptoms. But they are there, and they are not incompatible with cancer. Moreover, they don’t exclude it.

In an earlier study (Johnson-Laird et al., 2006), we showed that psychiatrists distinguish the two strategies as hyper-emotion theory predicts: corroboratory reasoning as the hallmark of patients suffering from various sorts of anxiety disorders, and refutatory reasoning as the hallmark of obsessive patients. Moreover, they do so even when the contents of vignettes, as in the examples above, provide no clue to the disorder. The aim of the present studies, however, was to test whether patients themselves recognize the two strategies (Experiment 1) and spontaneously use them in therapeutic sessions (Experiment 2). As there are still no studies that have investigated the origin of refutatory reasoning strategies in obsessive patients, in a third study (Experiment 3) we wanted to examine whether this form of reasoning actually stems from the (hyper-) guilt emotion.

## Experiment 1

The aim of this study was to verify whether patients themselves recognize their own reasoning strategy. It therefore used the same six matched pairs of vignettes used in the experiment with psychiatrists, including the pair in the Introduction, with the same contents. Hyper-emotion theory (e.g., Johnson-Laird et al., 2006) predicts that those suffering from anxiety will tend to see the corroboratory style of reasoning as being more similar to their own, whereas those suffering from obsessive-compulsive disorder will tend to see the refutatory style of reasoning as being more similar to their own.

### Method

#### Participants

The experiment tested two groups of patients: 18 patients with obsessive-compulsive disorder (male: 8; age: *M* = 32.7, *SD* = 7.5), and 20 patients with anxiety disorders (general anxiety disorder: 4, panic attack: 4, social phobia: 4, specific phobia: 4; hypochondria: 4; male: 14; age: *M* = 35.8, *SD* = 5.9). The two groups were similar in age (Mann–Whitney *U* = 167, *ns*) and educational level (obsessive patients: *M* = 14.4 years, *SD* = 1.7, anxiety patients: *M* = 14.2 years, *SD* = 1.7, Mann–Whitney *U* = 718, *ns*). Both groups were undergoing treatment at the Centre for Cognitive Psychotherapy in Rome but were not taking any medication. They were at the beginning of treatment and had been diagnosed through the Structured Clinical Interview and diagnosis for OCD and anxiety disorders in DSM-IV-TR (SCID; First et al., 1996).

#### Design, Materials, and Procedure

All participants read the same six matching pairs of vignettes as those employed in the earlier study of psychiatrists (see the two examples above; Johnson-Laird et al., 2006). Each pair illustrated the contrasting types of reasoning strategies (corroboratory vs. refutatory). The vignettes had been created based on the typical content of six psychological illnesses: Obsessive-Compulsive Disorder (of two types, one concerning contamination and the other concerning the checking compulsion), hypochondria, generalized anxiety, specific phobia, and paranoia (For translations of the vignettes from the original Italian language into English, see the Supplementary Appendix).

Patients were asked whether they wanted to take part in a study of the way people who ask for psychological help reason about certain crucial topics. They were told that there were no right or wrong answers and that it was their opinions that were of interest to the study. Before reading each vignette, the key question in the instructions they were given was this: *How similar is this vignette to how you reason when you think of what you are worried about because of your disorder?* The participants rated similarity on a seven-point Likert scale (from 0 = not similar at all, to 7 = absolutely similar). After having read each vignette, they were asked to describe the cues, if any, they used in evaluating the similarity. The vignettes were presented to the patients in a different single random order.

### Data Analysis

A research assistant, blind to the aim of the study, coded all paper data. Since our data were not normally distributed across groups, as assessed by Shapiro–Wilk’s test (all *P*s < 0.05), we employed non-parametric statistics.

### Results

In line with our hypotheses, we detected a critical interaction: the difference in selection between the refutatory and the corroboratory version was greater in obsessive patients than in anxious patients (Mann–Whitney *U* = 406, *p* < 0.001, η^2^ = 1.1).

As shown in Table 1, almost all the patients affected by OCD identified the refutatory vignettes as being more similar to the way they reasoned when worried. Moreover, their performance was at the ceiling of what was possible (98% of trials). However, they were unable to describe the indicators that they had used, and their judgments were quite rapid and intuitive. To further demonstrate that our obsessive patients identified with the refutatory vignettes more than they did with the corroboratory ones, for each patient and each pair of vignettes, we subtracted the rating they gave to the corroboratory vignette from the rating they gave to the refutatory one. We then computed a Wilcoxon test on the mean difference for each patient. In this way, we were able to confirm that the obsessive patients recognized refutatory vignettes as being more similar than corroboratory ones to their own reasoning (Wilcoxon, *z* = 3.73, *p* < 0.001, η^2^ = 0.08). Males and females were similar in their performance (Mann–Whitney *U* = 18, *ns*). By contrast, patients affected by other anxiety disorders felt the vignettes with corroboratory reasoning to be more similar to their type of reasoning when worried in 95% of trials; this was considerably higher than by chance (binomial test, *p* < 0.0001). Also, this group of patients was unable to describe the cues that they had used, and again their judgments were quite rapid and intuitive. Applying a similar procedure to that used for the obsessive participants, for each anxious patient and for each pair of vignettes, we subtracted this time the rating of the refutatory vignette from the rating of the corroboratory one. We thus further demonstrated that these patients recognize corroboratory vignettes as closer to their way of reasoning than refutatory vignettes (Wilcoxon, *z* = 3.95, *p* < 0.001, η^2^ = 0.08). No difference was found between the performance of male and female patients (Mann–Whitney *U* = 19, *ns*).

**TABLE 1:** Percentages of refutatory and corroboratory vignettes that obsessional patients and anxious patients rated as more similar to their own reasoning (>3 on the Likert scale) in Experiment 1.

	**Obsessive patients**	**Anxious patients**
	***N* = 18**	***N* = 20**
Refutatory versions (*n* = 6)	98%	55%
Corroboratory Versions (*n* = 6)	74%	95%

The vignettes were thus readily identifiable by both groups. This result supports the assertion of the theory that there are two characteristic ways of reasoning in patients. We therefore expected that OC patients and anxious patients would spontaneously reason in a refutatory and corroboratory form, respectively, during therapeutic sessions.

## Experiment 2

This study examined the spontaneous reasoning of obsessive patients and other anxious patients during therapeutic sessions. Our theory predicted that obsessive patients would spontaneously use the refutatory strategy more often when reasoning on topics typical of obsessions compared with when reasoning about other topics eliciting simply anxiety, e.g., work or relationships. In contrast, anxious patients should dominantly use the corroboratory strategy, both when reasoning about topics pertinent to their illness and when reasoning with other topics eliciting anxiety.

### Method

#### Participants

The experiment tested two groups of patients: 12 obsessive patients (Male: 6; age: *M* = 34.8, *SD* = 10.9) and 10 patients affected by panic attack (Male: 6; age: *M* = 33.9, *SD* = 9.5). The two groups did not differ in age (Mann–Whitney *U* = 58.5, *ns*) or educational level (obsessive patients: *M* = 14 years, *SD* = 2.1, anxiety patients: *M* = 14 years, *SD* = 1.5, Mann–Whitney *U* = 68, *ns*). Both groups were undergoing treatment at the Centre for Cognitive Psychotherapy in Rome but were not on any psychopharmacological treatment. They were at the beginning of treatment and had been diagnosed using the Structured Clinical Interview and diagnosis for OCD and panic disorder in DSM-IV-TR (SCID; First et al., 1996).

#### Design, Materials, and Procedure

We asked two colleagues in Rome, who had been trained in cognitive psychotherapy but were blind to the hypothesis being tested, to conduct this experiment. We asked them to instruct all the patients to put into words, during two different therapeutic sessions (i.e., thinking aloud) their ruminations and thoughts on (1) a topic that was pertinent to their illness. For example, for an obsessive patient, an episode of possible contamination, and for an anxious patient, an episode in which he had to use the elevator and (2) on a topic that was not pertinent to their illness. For example, for all patients, episodes regarding general worries about money or their job. These two therapists were asked to help patients during the task by posing such questions as:

*Put into words your thoughts while you are ruminating/thinking about the possibility of*….’ *“How are you reasoning about it?”“What are you telling yourself?”“What thoughts are crossing your mind?”*

The questions were the same for both the topics (pertinent topic vs. not pertinent topic to the illness). The patients were required to think aloud as they reasoned spontaneously while the therapist recorded what they said. The psychotherapists started to record audio the first time that a patient started to speak about a worry that crossed her/his mind. Two verbal reports were obtained from participants: one on a topic that was relevant to their condition, and the other on a non-relevant topic.

Two independent judges, both psychotherapists in Rome, who had also been trained in cognitive therapy and were blind to the hypothesis being tested, coded the pairs of recordings for the 22 patients. They were told to listen carefully as many times as they needed to in order to assign each recording to one of two mutually exclusive categories: patients using a refutatory reasoning strategy, and patients using a corroboratory reasoning strategy. They were given the following definitions of the two strategies:

– *Refutatory*: where the patient searches for counterexamples of the worst case under consideration.
– *Corroboratory*: where the patient searches only for examples of the worst case under consideration.

They also read two examples of each strategy, from two pairs of vignettes used in the earlier studies, each containing the same number of sentences. Where they disagreed in their judgments, which occurred in 3% of protocols, we asked a third judge (another psychotherapist in Rome) to make the final decision.

### Results

Table 2 presents an example of the refutatory reasoning of an obsessive patient and an example of the corroboratory reasoning of an anxious patient, both for a topic that was relevant for their illness.

**TABLE 2:** Two typical protocols describing problems relevant to the patient’s illness in Experiment 2, one from an obsessive patient using the refutatory strategy and one from an anxious patient using the corroboratory strategy.

**Obsessive patient**	**Anxious patient**
- I get off the bus, and I touch someone. I physically feel that my hand, or rather my fist, punched him. I think I hit him on the head. I think he could be dead. (*The patient focuses on his action, seeking to corroborate its negative consequences; he makes a transition to the emotion of guilt.)*	I am always thinking that I could die. I imagine dying. (*Individual focuses on a danger that leads to intense anxiety in patients*).
I looked back, but the bus was already gone. I keep thinking about it… If I had hit him, he would have at least reacted, he would have called for help, he would have beaten me. (*He tries to infer counter-examples to the negative outcome of his having harmed the other person.*)	Yesterday, I remembered that my grandfather suffered from two heart attacks, and I often feel pain in my left arm. (*He searches for evidence confirming his hypothesis.*)
Yes, but it all happened so fast. But people would have said something, they would have stopped me. *(He searches again for counter-examples to the negative outcome.)*	Moreover, last week I moved, and so I have also carried many heavy boxes. I was very tired and stressed. I felt tachycardia, and my heartbeat so fast even when I was driving home. (*He searches for further corroboratory evidence.*)
What if no-one noticed it until it was too late? (*He thinks again of a corroboration.)*	I know that my doctor thinks I’m exaggerating, but I couldn’t ignore what I felt. I kept thinking: it could be a real heart attack this time. (*He continues to corroborate the hypothesis.*)

*Comments in parentheses highlight the crucial cues to the strategy.*

The Cohen’s kappa correlation coefficients between the two judges for the two reasoning strategies (refutatory or corroboratory) were 0.83 for reasoning related to patients’ illness and 0.65 for reasoning about other topics. Overall, Cohen’s kappa for the reliability of their judgments was 0.73, which reflects a good agreement (Fleiss, 1981). For the few protocols on which they disagreed, the third judge cast the deciding vote.

As shown in Table 3, obsessive patients tended to use a refutatory strategy and anxious patients tended to use a corroboratory strategy (Fisher–Yates exact test: *p* < 0.05, η^2^ = 0.05) when they reasoned on a topic the was relevant for their illness. In contrast, when the two groups of patients thought about topics other than their illnesses, both of them tended to use the corroboratory strategy (Fisher–Yates exact test: *p* > 0.5).

**TABLE 3:** Frequencies of protocols reflecting a refutatory reasoning strategy and those reflecting a corroboratory reasoning strategy on a topic pertinent to the obsessive disturb or anxiety illness in Experiment 2.

	**Relevant topics (*n* = 22)**		**Non-relevant topics (*n* = 22)**	
		
	**Refutatory**	**Corroboratory**	**Refutatory**	**Corroboratory**
	**strategies**	**strategy**	**strategies**	**strategy**
OC patients	10	2	1	11
(*N* = 12)				
Anxious patients	1	9	0	10
(*N* = 10)				

## Experiment 3

A number of studies have previously demonstrated that (hyper-) anxiety is responsible for the corroboratory pattern of reasoning (see de Jong et al., 1998). To date, no study has investigated the origin of the refutatory reasoning strategy in obsessive patients. In line with the hyper-emotion theory, with this study, we wanted to examine whether the latter form of reasoning actually stems from the (hyper-) guilt emotion. With this aim, we used two different vignettes: one in which the protagonist was guilty and responsible for the negative outcome (see below: vignette 1), and one in which a third person was responsible and guilty for the outcome (see below: vignette 2). According to the idea that obsessive symptomatology is based on the threat of being guilty, assessed as being imminent and the goal being to prevent it, we hypothesized that obsessive patients would use the refutatory strategy more than the Better Safe than Sorry (i.e., corroboratory) strategy, more so in scenarios in which they were guilty concerning a negative outcome (see vignette 1) than in scenarios in which others were guilty of the same outcome (see vignette 2, reported below), and more than patients suffering from other anxiety disorders.

### Method

#### Participants

The experiment tested two groups of patients: 13 obsessive patients (Male: 8; age: *M* = 33.5, *SD* = 7.7) and 11 patients affected by panic attack (Male: 7; age: 32.6, *SD* = 7.4). The two groups were similar in age (Mann–Whitney *U* = 66.5, *ns*) and educational level (obsessive patients: *M* = 14 years, *SD* = 2.1, anxiety patients: *M* = 14.8 years, *SD* = 1.7, Mann–Whitney *U* = 52, *ns*). Both groups were undergoing treatment at the Centre for Cognitive Psychotherapy in Rome but were not on any psychopharmacological treatment. They were in the starting phase of treatment and had been diagnosed using the Structured Clinical Interview and diagnosis for OCD and panic disorder in DSM-IV-TR (SCID; First et al., 1996).

#### Design, Materials, and Procedure

To recruit these two groups of patients, at the end of the first session of their clinical assessment, patients were asked whether they wanted to take part in a study on the way people reason about certain crucial topics. They were told that there were no right or wrong answers and that we were only interested in their opinions. Four colleagues in Rome and in Palermo, who had been trained in cognitive psychotherapy and who were blind to the hypothesis being tested, were asked to carry out the experiment. Patients were instructed to read two short vignettes, each leading to a negative outcome: one described a situation in which the protagonist of the story could be responsible or guilty for the negative outcome, while in the other, the possible culprit was a third person. They were then asked to reason about both stories, writing down their thoughts in order to reassure themselves beyond any reasonable doubt about the negative outcome.

The story concerning the culpability of the protagonist was, for example (vignette 1):

Imagine that it’s Sunday afternoon and I’m with my niece. I’m playing with her on the sofa when my nose starts itching, and I sneeze. I don’t care and keep on playing with her. Later, it strikes me that my niece might be sick because of my sneeze. It would be because of my carelessness. I should have been more careful.

The story concerning the culpability of a third person was, for example (vignette 2):

Imagine that I take my niece to the kindergarten. I see her playing with other kids and the teacher. As I approach them, the teacher’s nose starts itching, and she sneezes several times. She doesn’t care and keeps on playing with my niece. Later, it strikes me that my niece might be sick because of her teacher’s sneeze. It would be because of her carelessness. She should have been more careful.

After having read each story, all participants were told:

Try to reassure yourself about this possibility, beyond any reasonable doubt. Write all the thoughts that come into your mind.

The participants in each group read the two stories in a different random order and in two different therapeutic sessions.

Two independent judges, both psychotherapists in Palermo who were blind to the hypothesis being tested, categorized the two protocols from each participant in terms of whether they exhibited a refutatory or a corroboratory reasoning strategy (see Study 2 for their instructions). Where they disagreed on their judgments, a third judge (another psychotherapist in Palermo) cast the deciding vote.

### Results

Table 4 shows two typical protocols of the two sorts of reasoning from two representative obsessive patients. Agreement between the two judges regarding the two forms of reasoning (corroboratory vs. refutatory) was 0.73 for the stories concerning the protagonist’s guilt and 0.83 for the stories concerning another person’s guilt (Cohen’s kappa = 0.78). They disagreed on only four protocols.

**TABLE 4:** Two typical protocols of the two sorts of reasoning from two representative obsessive patients, reasoning about the story eliciting guilt (refutatory strategy) and the story describing another’s guilt (corroboratory strategy) in Experiment 3.

**Refutatory strategy for story eliciting protagonist’s guilt**	**Corroboratory strategy for story describing other’s guilt**
– Surely it doesn’t depend on that, but if I had a cold, it is. The mere fact that I sneezed made the air full of germs. (*The participant corroborates the negative outcome.)* – Maybe the window was open. If so, the germs could have gone out (*to refute the negative outcome*). – Nevertheless, they could have contaminated the kid; they could have been everywhere in the air (*to corroborate the negative outcome*). – Surely it was a coincidence. Maybe she already had a cold (*a refutation).* – But what if this is not the case? (*A corroboration.*)	The teacher had a cold, and she sneezed. So, the probability that she contaminated my niece is very high. *(The participant corroborates the negative outcome.)* Moreover, she was playing with the kid *(a corroboration).* So, the air was contaminated. I cannot see how it could not have been contaminated, although they were in the playground *(a corroboration).* Moreover, they were so close *(a corroboration).* So, if my niece fell ill, she was truly contaminated by the sneezes *(a corroboration).*

*Comments in parentheses highlight the crucial cues to the strategy.*

As shown in Table 5, obsessive patients were prone to use a refutatory strategy with a story concerning their own guilt more than were anxious patients (Fisher–Yates exact test: *p* < 0.05, η^2^ = 0.04), while, with the story describing another person’s guilt, they tended to reason in a corroboratory way, as did the anxious patients (Fisher–Yates exact test: *p* > 0.5).

**TABLE 5:** Frequencies of protocols reflecting a refutatory reasoning strategy and those reflecting a corroboratory strategy in obsessive and anxious patients trying to refute the outcome that the protagonist in the story might be guilty in Experiment 3.

	**Story eliciting**		**Story describing**	
	**protagonist’s guilt**		**another’s guilt**	
		
	**Refutatory**	**Corroboratory**	**Refutatory**	**Corroboratory**
	**strategy**	**strategy**	**strategy**	**strategy**
Obsessive patients	12	1	2	11
(*N* = 13)				
Anxious patients	4	7	1	10
(*N* = 11)				

## Discussion

With our studies, we have demonstrated that individuals affected by psychological disorders produce characteristic reasoning strategies that depend on the hyper-emotion elicited by a threat. In particular, in our first study, we showed the ability of anxious patients and obsessive patients to recognize the refutatory and the corroboratory patterns of reasoning, respectively, as more similar to their own, regardless of the content of the inference. In the second study, we further demonstrated that obsessive patients spontaneously produced the refutatory pattern of reasoning, while other anxious patients produced the corroboratory pattern when thinking about topics that were condition-relevant. Finally, in accordance with hyper-emotion theory (Johnson-Laird et al., 2006), we showed that corroboratory reasoning was elicited by anxiety, while a refutatory form of reasoning stemmed from the (hyper-) guilt emotion (Experiment 3). The former result is in line with the wider literature showing that, in the face of exposure to a threat eliciting anxiety, individuals suffering from hypochondria or other anxiety disorders are inclined to focus on the danger and to search for examples confirming it (e.g., de Jong et al., 1998; Gilbert, 1998).

The latter finding is significant because, as anticipated above, no other study has investigated either the refutatory form of reasoning or its origin in obsessive patients, except for the study by Giele et al. (2011). The obsessive-like step-by-step reasoning is the only example of a strategy that is comparable to our refutatory pattern of reasoning. It is worth noting that both their and our studies demonstrate that this form of reasoning has a paradoxical effect. The obsessive-like creation of small steps leading from an innocuous situation to a catastrophic consequence increases the plausibility of the feared outcome, potentially maintaining the obsessive condition. Such reasoning begins with thoughts about the possible danger (Johnson-Laird et al., 2006), whereby patients try to defend against this possible danger and attempt to consider the situation in a comprehensive way. As a consequence, the obsessive patient begins to make a series of steps toward this self-created danger. However, this strategy has the ironic effect of strengthening the belief that the feared event will actually happen. Our findings appear to add to the growing list of studies showing that the effects of reasoning in psychological disorders run counter to the real intentions of patients: the safety strategies used by patients are counterproductive and lead to a decrease, instead of an increase, in confidence that there will be no negative outcome.

### Limitations and Future Research

Our studies have several limitations. Although our aim was to investigate the reasoning strategies of patients affected by certain psychological disorders, one limitation is that we did not have any healthy control group. Therefore, future studies should investigate what style of reasoning non-clinical individuals use or what style of reasoning, if any, they recognize as being their style.

A second limitation, again pertaining to all of our studies, is that we did not include at least a clinical control group characterized by an emotion other than either anxiety or guilt – for example, depressed people with sadness. Hyper-emotion theory (e.g., Johnson-Laird et al., 2006) predicts that the reasoning strategy of depressed people will be different from the two groups analyzed in this paper. Such individuals pay particular attention to a person or situation assessed as being lost, and they feel intense sadness about this. Individuals attempt to infer the more positive conclusion that the loss is not permanent (positive hypothesis) and try to corroborate it. But the more they pay attention to the lost person or entity, the higher their standards for what would be acceptable as a substitute are set. As a consequence, they infer that the loss is irreplaceable (falsifying the positive hypothesis) (see also Mancini and Gangemi, 2015). According to this, we could expect that depressed patients would recognize neither of our two reasoning strategies (corroboratory and refutatory) as similar to their own and would reason in a different way. Future studies should thus investigate other styles of reasoning, in order to verify whether and how changing the emotion changes the reasoning strategy as well.

A third critique is pertinent to Experiment 3, specifically. Here, we used the same experimental procedure we had used in earlier experiments (see Johnson-Laird et al., 2006; Gangemi et al., 2013), where we asked participants to read stories whose contents were designed to elicit guilt in the protagonist. Skeptics may argue that how emotions explain reasoning in such an experiment is something of a mystery. However, hyper-emotion theory does propose an explanation. It states that emotions stimulated by the topic of the story (the guilt of the protagonist vs. the guilt of other persons) lead individuals to be motivated to reason in a way pertinent to their source in order to reassure themselves about the damage. This effect, together with the standard inferential ability, yields the pattern of inferences in our experiment. However, future studies should further verify whether guilt actually elicits the refutatory strategy and anxiety elicits the corroboratory strategy in this study, for example, by assessing the level of both the emotions before and after having read each story with a manipulation check questionnaire.

### Clinical Implications

This study may have some clinical implications. In general, if hyper-emotion theory is correct, then there are transitions from normal life emotions to abnormal ones in psychological disorders. Therefore the therapeutic goal should have as its focus the disengagement of these transitions and of patterns of inference that would otherwise boost the aberrant emotions. For example, it appears that when patients acquire the ability to accept the possibility of being guilty, there is a decrease in obsessive symptoms, even when guilt acceptance is not related to the patient’s symptomatic domain (Cosentino et al., 2012). Moreover, it is common for patients to experience a feared situation, quickly imagine a catastrophic outcome, and to be engaged in one of the two forms of reasoning. A sort of meta-cognitive intervention focused on leading patients to become aware of the way they reason on the disorder-related threat could be very helpful. Explaining, for example, that the refutatory reasoning in the case of catastrophes feared by OCD subjects will be counterproductive may actually be effective.

Finally, in certain cases, the steps leading from a neutral situation to a catastrophic outcome are not properly elaborated by the anxious or obsessive patient or may appear particularly implausible to the therapist. Therapists might, in such cases, attempt to test these catastrophic scenarios by asking how exactly the patient imagines that the particular transitions could take place (e.g., “how exactly might HIV be transmitted from the door to the hat?”) Such therapeutic intervention may be risky, in the sense that it could foster the process that is examined in this paper: it may paradoxically increase the plausibility of the feared outcome.

## Conclusion

In sum, patients develop characteristic strategies of reasoning that depend on the (hyper-) emotion elicited by the threat: anxious patients make corroboratory inferences, adducing only evidence that confirms the risk (*corroboratory strategy*), while obsessive patients make refutatory inferences, adducing counter-examples disconfirming the risk (*refutatory strategy*). There is a paradoxical effect of these reasoning strategies that contributes to the maintenance of psychological disorders, systematically leading to the confirmation of the dysfunctional beliefs that are central to these illnesses.

## Data Availability Statement

The datasets generated for this study are available on request to the corresponding authors.

## Ethics Statement

The studies involving human participants were reviewed and approved by the Associazione di Psicoterapia Cognitiva, Rome, Italy. The patients/participants provided their written informed consent to participate in this study.

## Author Contributions

AG and FM conceived and designed the experiments and analyzed the data. AG and KT collected the data. AG, FM, and KT wrote the manuscript.

## Conflict of Interest

The authors declare that the research was conducted in the absence of any commercial or financial relationships that could be construed as a potential conflict of interest. The handling Editor declared a shared affiliation, though no other collaboration, with one of the authors AG at the time of review.
