# Supplementary material for: Two Reasoning Strategies in Patients With Psychological Illnesses
Source: Front Psychol. 2019 Oct 22;10:2335. doi: 10.3389/fpsyg.2019.02335 (PMC6817569; doi:10.3389/fpsyg.2019.02335)
Supplement: Supplementary file 1 [file Presentation_1.pdf]

Appendix

The vignettes used in Experiment 1, translated from Italian. They were formerly used by Johnson-Laird, Mancini and Gangemi (2006).

A1. A vignette in the refutatory form of reasoning of a person suffering from obsessive-compulsive disorder, with content concerning illness:

I'm afraid of the little pain that I'm feeling in my abdomen, on the same side as my liver. It could be a symptom of cancer, a liver cancer. I remember an uncle of mine who died from liver cancer after suffering a lot. But he was in his eighties and I'm thirty, and a liver cancer at my age is rare. On the other hand, it's not impossible. Moreover, I think I look unhealthy; my tongue is dirty; sometimes my mouth tastes bitter. I look pale, and I could have anaemia. Of course, these are common symptoms and they could be trivial. I have had them many other times. But they are there, and they are not incompatible with cancer. Moreover, they don't exclude it. My doctor prescribed several tests for me and the results were all negative. But, the results could be those of another person—sometimes laboratories mix up test-tubes, or the secretary makes a mistake writing the patient's name or puts one person's results in somebody else's folder. A mistake can always occur. The laboratory may be very professional, but there cannot be a one-hundred-percent guarantee that it didn't make a mistake. Moreover, I am the main person responsible for my own health. Imagine how I would feel if I really had cancer and left it too late. The best thing to do is to go back to my doctor.

A2. A vignette in the corroboratory form of reasoning of a person suffering from, e.g. hypochondria, with content concerning illness:

I'm afraid of the little pain that I'm feeling in my abdomen, on the same side as my liver. It could be a symptom of cancer, a liver cancer. I remember an uncle of mine who died from liver cancer after suffering a lot. In the beginning, his symptoms were the same as mine: he had a similar stomach ache. He didn't care, and the doctors told him that he wasn't ill. But meanwhile, the cancer was spreading. Now, in the same way, the cancer may be spreading in my abdomen. Indeed, my symptoms seem to have become worse during the last few weeks. Nobody believes me, and nobody takes me seriously. When they do start to treat me, it will be too late! Moreover, I think I look unhealthy; my tongue is dirty; sometimes my mouth tastes bitter. I look pale, and I could have anaemia. What a trauma it will be for me and my family when the cancer is correctly

diagnosed and it’s too late! Afterwards, my life will be one of suffering, drugs, medical tests, checks and surgical operations. The best thing to do is to go back to my doctor.

B1. A vignette, in the refutatory form of reasoning, of a person suffering from obsessive-compulsive disorder (OCD), with content concerning contamination:

I touched a magazine with a close-up of Rock Hudson, a famous gay actor dying of AIDS, on the cover page and I may have caught AIDS. The photographer was near RH and the photo is a close-up. But AIDS is not contagious simply by being close; there has to be intimate contact. Yet, there may have been intimate contact, for all I know. The photographer could have been gay. In fact, it seems unlikely that there could have been any intimacy in a hospital ward with a seriously ill patient, but I wasn’t there so how can I rule it out? They might simply have kissed, and no-one has ever been able to find out with complete certainty whether a kiss is dangerous. The photographer, being a professional, certainly developed the film and printed the photos himself and so may have contaminated them. Indeed, he may not have washed his hands after a sexual act or he may have had a cut on his hand that bled and actually contaminated the photos and the negatives. But even if he had contaminated the negatives and photos, viruses die! Yes, but some may have survived; I cannot be sure they were all killed and so some may have survived. The contamination may even have occurred when the photographer was taking the photos and negatives to the printers of the magazine. The photos and negatives were then received by a printer who may himself have contaminated them. This scenario seems absurd too, but you cannot actually be certain that the printer was not contaminated. If this was the case, then you cannot rule out the possibility that the printing press was contaminated, and that the printed copies of the magazine, and thus the copy I am holding too, was contaminated too. By touching it I may have been contaminated myself.

B2. A vignette in the corroboratory form of reasoning of a person suffering from, e.g. general anxiety disorder, with content concerning contamination:

I touched a magazine with a close-up photo on the cover page of Rock Hudson, a well-known gay actor who was dying of AIDS and I may have caught AIDS. The photographer was near RH and the photo is a close-up. As they were close together they may have touched each other, perhaps even in an intimate fashion; for instance, they may have kissed. The photographer could have been gay. He may have caught AIDS in this way. The photographer, being a professional, certainly developed the film and printed the photos himself and thus may have contaminated them.

Indeed, he may not have washed his hands after a sexual act or may have had a cut on his hand that bled and actually contaminated the photos and the negatives. The contamination may even have occurred when the photographer was taking the photos and negatives to the printers of the magazine. The photos and negatives must have been touched by the printers printing the magazine; and so the virus may have also contaminated the printers who certainly touched the photo, perhaps with dirty or injured hands, and then contaminated the printing press and the copies of the printed magazine including the copy I am holding. By touching it I may have been contaminated myself.

C1. A vignette in the refutatory form of reasoning of a person suffering from obsessive-compulsive disorder (OCD), with content concerning threat:

I've just left the house and the thought comes to me that I have left the gas on. I remember turning it off and checking it but I am not completely sure, and maybe I am getting confused with the check I made the other day. Perhaps I should go back and check. The gas might leak! I know there are safety valves but they are not 100% secure, and all you need is a tiny spark for everything to explode. It is true that I think I will be back in 20 minutes, but that could be long enough for an explosion. And if there is an explosion it might destroy the whole building as well as the neighbouring buildings, killing dozens of people! Of course, catastrophes are rare events but this one is possible. I'd better to go back and check.

C2. A vignette in the corroboratory form of reasoning of a person suffering from, e.g. general anxiety disorder, with content concerning threat:

I've just left the house and the thought comes to me that I have left the gas on, as has already happened to me once and as happened to the Foligno family whom I saw yesterday evening on the news. Nothing happened to me the other time, but their house blew up. The poor things ended up in a charity home! A month ago, the man came to check the meter and said that I should be very careful because for this particular period—I don't know for what technical reason—the gas company is delivering a particularly inflammable and odourless gas. And so the neighbours might not notice a possible leak. I'd better go back and check.

D1. A vignette in the refutatory form of reasoning of a person suffering from obsessive-compulsive disorder (OCD), with content concerning spiders:

I have to go down to the cellar but I am afraid of finding one of those big, hairy, poisonous and aggressive spiders. Spiders prefer damp cellars and one might prefer to hide under crates like the ones I have to move in order to look for some old documents that I need. However, I recall that spiders are not dangerous, although perhaps some South American species are. Yes, but we are not in the tropics; however, it is also possible that some inhabitants of the building might have kept some spiders as pets—perhaps the young fellow who is a steward and who may also travel to South America. He would keep them in a case and certainly not free in the house! But what if the case was broken, and a spider, one of those big, hairy, poisonous ones, escaped and the steward failed to catch it or was away travelling? And suppose that the spider has hidden in my cellar—perhaps right under the crates with my documents. I think I'd better not go down to the cellar.

D2. A vignette in the corroboratory form of reasoning of a person suffering from, e.g. a specific phobia, with content concerning spiders:

I have to go down to the cellar but I am afraid of finding one of those big, hairy, poisonous and aggressive spiders. I have read that spiders prefer damp cellars, where they hide under crates, just like the ones I have to move in order to find some old documents that I need. How awful! I would certainly be petrified with fear. Above all, I would be alone and there would be no-one to help me as the spider came nearer. Moreover, on the first floor there is a shop that sells aquaria, tropical fish, reptiles and insects of the most exotic, large, and odd variety. A month ago, when I leant out to ask about the condominium meeting, I remember there was a glass case with a horrible big hairy spider from South America, just like the one I saw later in an encyclopaedia that said it was poisonous. Now that I think of it, I heard the doorman say a few weeks ago that the new Philippine shop assistant had broken some glass cases containing insects, which then escaped. Just think if one of them was under the crates! Perhaps I'd better forget about going down into the cellar.

E1. A vignette in the refutatory form of reasoning of a person suffering from obsessive-compulsive disorder (OCD), with content concerning threat:

There are a lot of Arabs in town, many of whom are young and always have scowling expressions on their faces that I would describe as resentful. But there could be a thousand reasons why they have those expressions. Yes, but among those various reasons could be a hatred of the West. There might also be terrorists among them. It is true that there is police surveillance, but I read in the paper that terrorists are sometimes 'sleepers' for years, and so escape police attention completely. It is not inevitable that an attack will be carried out near my son's school. Yes, but

> the school my son goes to is near a very busy shopping mall, an ideal place for an attack—hard to keep under surveillance and packed with people. And my son has to go past it to get to school. Yes, these are very general reasons for concern; but on the other hand, the fact that they're general doesn't mean that they are not real. The other day I was passing by there, and now that I think of it I got the impression that there were some Arabs nearby in a van. They may have been Italians, but I cannot rule out that they were Arabs. I think I've seen that same van in the neighbourhood two or three other times. Perhaps they simply work around there; yes, but they might also have been doing reconnaissance before an attack. It is a fact that to plan an attack it is necessary to carry out numerous inspections. How could I possibly rule this attack out? The van didn't seem to be solid enough to transport many explosives. However, it did closely resemble the one that the TV reporter said had been used in an attack. Yet, goodness knows how many vans of this kind there are. But, why couldn't it have just been that one? Of course, there'd be a tremendous explosion because there'd be a huge quantity of explosives. My son would be blown to pieces like all those poor children I saw on TV.

E2. A vignette in the corroboratory form of reasoning of a person suffering from, e.g. general anxiety disorder, with content concerning threat:

> There are a lot of Arabs in town, many of whom are young and always have scowling expressions on their faces that I would describe as resentful. There might be terrorists among them; among other things, I read in the paper the other day that terrorists can sometimes be 'sleepers' for years, and so escape police attention completely. Then, the school my son goes to is near a very busy shopping mall, an ideal place for an attack—hard to keep under surveillance and packed with people. And my son has to go past it to get to school. The other day I was passing by there and, now that I think of it, I got the impression that there were some Arabs nearby in a van. Then, I think I later saw the same van in the neighbourhood another two or three times. It may have been doing reconnaissance before an attack. They may use the van to transport explosives. It did closely resemble the one that the TV reporter said had been used in an attack. There'd be a tremendous explosion because there'd be a huge quantity of explosives. My son would be blown to pieces like all those poor children I saw on TV.

F1. A vignette in the refutatory form of reasoning of a person suffering from obsessive-compulsive disorder (OCD), with content concerning mickey-taking (persecution)

And if my students take the mickey out of me? Of course, I cannot prove it as they could make fun of me behind my back without me seeing them. But, why would they waste their time on me? Yes, but it is a fact that students are cruel to teachers and like to have fun at their expense. I remember that when I was at high school there was a teacher, probably gay, and my friends and I had fun at his expense for years. And I remember how my friends made fun of him as soon as his back was turned. Yes, but I would have noticed! In actual fact, however, last week I saw a little group laughing amongst themselves as I came into the lecture room. But they may have been laughing over something else, possibly a joke. Yet, I cannot be sure, and indeed what grounds do I have for ruling out this possibility? Perhaps they were not making fun of me on that occasion, but they could have done it without me realising it when I was preoccupied or thinking of something else. Perhaps I simply cannot remember properly.

F2. A vignette in the corroboratory form of reasoning of a person suffering from, e.g. paranoia, with content concerning mickey-taking (or persecution):

As soon as I entered the lecture room I saw the students chatting together and among their almost imperceptible words I caught the word ‘queer’. They were taking the mickey out of me. Did you see how they were sniggering yesterday at the lecture and in the corridors as I was going past? Then the other day one of them was sitting in the first row right in front of me; I was about to start the lecture and he addressed the student next to him in an effeminate tone of voice. He was clearly referring to me. It is a known fact that students are cruel to teachers and like to have fun at their expense. I remember that when I was at high school there was a teacher, probably gay, and my friends and I had fun at his expense for years. And I remember how my friends made fun of him as soon as his back was turned. Of course they are taking the mickey out of me!
